# Inositol and In Vitro Fertilization with Embryo Transfer

**DOI:** 10.1155/2017/5469409

**Published:** 2017-02-28

**Authors:** G. Simi, A. R. Genazzani, M. E. R. Obino, F. Papini, S. Pinelli, V. Cela, P. G. Artini

**Affiliations:** Division of Obstetrics and Gynecology Oncology, Department of Clinical and Experimental Medicine, University of Pisa, Pisa, Italy

## Abstract

Recently, studies on inositol supplementation during in vitro fertilization program (IVF) have gained particular importance due to the effect of this molecule on reducing insulin resistance improving ovarian function, oocyte quality, and embryo and pregnancy rates and reducing gonadotropin amount during stimulation. Inositol and its isoforms, especially myoinositol (MYO), are often used as prestimulation therapy in infertile patients undergoing IVF cycle. Inositol supplementation started three months before ovarian stimulation, resulting in significant improvements in hormonal responses, reducing the amount of FSH necessary for optimal follicle development and serum levels of 17beta-estradiol measured the day of hCG injection. As shown by growing number of trials, MYO supplementation improves oocyte quality by reducing the number of degenerated and immature oocytes, in this way increasing the quality of embryos produced. Inositol can also improve the quality of sperm parameters in those patients affected by oligoasthenoteratozoospermia.

## 1. Introduction

Despite 30 years of history, assisted reproduction technologies (ART) still present many challenges in order to identify factors and predictors of success.

It is well known that oocyte quality is the main factor determining chance of pregnancy and that poor quality is an obstacle for successful in vitro fertilization results. It has become increasingly clear that the follicular microenvironment of a human oocyte is a crucial factor for its developmental competence [1].

Through the years, many studies have been proposed to find strategies, drugs, or compounds such as antioxidant drugs and supplementation with vitamins or hormones able to improve oocyte quality and embryo quality [2].

Recently, studies on inositol supplementation during in vitro fertilization program (IVF) have gained particular importance due to the effect of this molecule on reducing insulin resistance improving ovarian function, oocyte quality, and embryo and pregnancy rates and reducing gonadotropin amount during stimulation [3]. Inositol and its isoform, especially myoinositol, find their application as prestimulation therapy in polycystic ovary syndrome (PCOS) patients undergoing IVF cycle and, recently, also in all kinds of infertile patients such as poor responders [4].

Studies demonstrate that the use of inositol in male patients affected by oligoasthenoteratozoospermia can improve sperm cell parameters and consequently the impact of fertilization rate and embryo quality leading to high percentage of pregnancy [5].

## 2. Inositol

Inositol (cyclohexanehexol) is a cycle polyol commonly referred to as a B vitamin, although not a true vitamin. It is widely distributed in human tissue and cells, and it is a precursor for phosphorylated compounds known as phosphoinositides which are involved in signal transduction through membrane receptor stimulation and other secondary messengers including diacylglycerol (DAG) and inositol triphosphate (IP3) that can be located at the inner or outer side of membrane and are involved in insulin transduction signaling. DAG activates protein kinase C (PKC) and IP3 activates intracellular calcium (Ca2+) release, an essential step in oocyte maturation and so of fertilization process. There are nine inositol stereoisomers, and myoinositol is the most represented in cellular content [6]. All stereoisomers act as mediator of insulin action inside the cell [7]: myoinositol (MYO) and D-chiro-inositol (DCI) are inositol-containing phosphoglycan (IPG) mediators, generated by hydrolysis of glycosylphosphatidylinositol that inhibits cyclic AMP-dependent protein kinase (the first) and activates pyruvate dehydrogenase (the second one) [8].

MYO has been shown to influence different pathways at both ovarian and nonovarian levels. MYO is an important constituent of follicular microenvironment and it plays a determinant role in both nuclear and cytoplasmatic oocyte development [9], being also a precursor of phospholipids, which are responsible for the generation of important intracellular signals oocytes such as release of cortical granules, inhibition of polyspermy, and resumption of meiotic process [10]. Furthermore, MYO seems to significantly modulate steroidogenesis by acting through an insulin-independent pathway that involves cytoskeleton rearrangements [11].

On the contrary, DCI alone is not able to make significant improvements in the ovarian cell functions, as its beneficial effects are mainly confined to the nonovarian tissue in which it may significantly inhibit the negative cellular consequences of hyperinsulinemia. However, both inositol isomers can be effectively used in the management of PCOS patients in a ratio corresponding to their physiological plasma ratio (40 : 1). This seems to exert a synergistic effect according to a multitargeted design [12].

According to DCI ovary paradox theory, an increase of epimerase function in the ovaries causes an increase of DCI level associated with a local MYO deficiency and poor oocyte quality [13] with a negative effect in FSH stimulation and in ovulation [14]. Finally, some studies observed that high dosage of DCI administration may damage oocytes [15].

## 3. Inositol and In Vitro Fertilization

During the last decades, researches have focused on the role of the two major inositol stereoisomers, MYO and DCI, in particular on the effects of the first on oocyte quality. Among the causes of infertility, PCOS patients undergoing ovarian stimulation are subjected to an increased risk of in vitro fertilization failure due to poor oocyte/embryo quality and/or risk of ovarian hyperstimulation syndrome (OHSS). One of the goals of ovarian stimulation in PCOS is to recover an adequate number of mature oocytes avoiding OHSS. Clinical trials show that MYO supplementation started three months before the onset of ovarian stimulation results in significant improvements in hormonal responses, reducing the international unit (IU) of FSH needed to an optimal follicular development and estradiol levels at the day of ovulation trigger; this leads to a reduced risk of ovarian hyperstimulation syndrome (OHSS) and a lower number of canceled cycles. As shown by growing number of trials, MYO supplementation positively correlates with the number of oocytes retrieved and more importantly with the good quality of these. This means a reduction in the number of degenerated and immature oocytes, with consequently increased quality of embryos produced after fertilization [3, 16–18].

This result, obtained in both PCOS patients and non-PCOS women, is confirmed in the observation of Chiu et al. [19], who found that the amount of gonadotropin used for ovarian stimulation during IVF cycles is reduced in women whose follicular fluid contains higher levels of MYO. For these reasons, myoinositol supplementation appears really promising in ART and its effect has also been tested in non-PCOS patients undergoing fertility treatment in ART.

## 4. Inositol and PCOS in IVF

Polycystic ovary syndrome is one of the most common causes of infertility affecting 5–10% of females in reproductive age [20]. Typically, PCOS is characterized by hyperandrogenism (extremely variable in its occurrence), ovarian dysfunction with chronic anovulation and irregular menstrual cycle, polycystic ovaries at ultrasound evaluation, and dermatological problems such as acne, hirsutism, and seborrhoea [21].

Nowadays, a lot of trials show the importance of impaired insulin sensitivity as reason for many PCOS symptoms [22]. It has been hypothesized that an abnormal insulin signal transduction may cause insulin resistance, which induces anomalous ovarian steroidogenesis [20, 21]. Current therapies try to improve insulin resistance in order to reduce hyperinsulinemia resulting in metabolic and ovary function improvement. Insulin sensitizers, such as metformin, represent first line of therapy, but recently also inositol and its stereoisomers, myoinositol and D-chiro-inositol, have gained a lot of importance. MYO and DCI improve metabolic and endocrine function in PCOS patients; in particular, they determine a reduction in systolic arterial pressure and a reduction of LH/FSH ratio, and in addition they reduce circulating androgen and prolactin levels, increasing insulin action and sex hormone-binding protein levels [23]. Studies underlined the importance of myoinositol in oocyte differentiation and inositol ability to improve fecundation with in vitro fertilization techniques in women with PCOS compared to supplementation with D-chiro-inositol, which can damage oocyte if administered at high dosage [13, 15].

Recent trials note that women with PCOS respond to DCI with an increase in ovarian activity and menstrual frequency [24, 25]. However, subsequent studies demonstrated that MYO is more active than DCI [3, 26, 27] leading to regular menstrual cycles [24, 27] and to improvement of oocyte quality [28].

MYO supplementation during IVF cycle in PCOS patients increases oocyte quality [3, 16, 17] and embryo quality [29] and consequently implantation rate [4]. A recent analysis of follicular fluid in PCOS patients shows a 500-fold reduction in the amount of MYO, associated with an increase of insulin resistance, hyperinsulinemia, and luteinizing hormone levels [30].

Our recent study evaluates the effects of MYO administration on hormonal parameters in PCOS. 50 overweight PCOS patients undergo hormonal evaluations and an oral glucose tolerance test (OGTT) before and after 12 weeks of supplementation with myoinositol. Patients are divided into two groups: one treated with MYO 2 g and folic acid 200 *μ*g daily (Group A) and the other one receiving only folic acid 400 mg (Group B-controls). Ultrasound examinations and Ferriman-Gallwey score are also performed. We note that after 12 weeks of MYO administration plasma LH, PRL, T, insulin FSH result/FSH resulting significantly reduced. Insulin sensitivity, expressed as glucose-to-insulin ratio and HOMA index that indicate the insulin resistance ([basal glucose] × [basal insulin]/22.5), results significantly improved after 12 weeks of treatment. Menstrual cyclicity is restored in all amenorrheic and oligomenorrheic patients, while no changes occurred in patients treated with folic acid. After twelve weeks of treatment, an IVF cycle is performed for each patient: in the study group, the duration of stimulation is lower than in control group (11.5_0.8 versus 12.6_1.1; p¼0.002) and also rFSH units used are fewer. 17b–E2 levels (1839_520 versus 2315_601; p50.002), evaluated at the ovulation trigger day, are lower in the MYO group. Moreover, pregnancy rate (bHCG positive) is statistically significantly higher in the treated group (60% versus 32%; p50.05) [9].

Recently, Pacchiarotti et al. tested the synergistic effect of myoinositol and melatonin in IVF protocols with PCOS patients in a randomized, controlled, double-blind trial. They randomly divided five hundred twenty-six PCOS women into three groups: controls (only folic acid: 400 mcg), Group A (a daily dose of myoinositol: 4000 mg, folic acid: 400 mcg, and melatonin: 3 mg), and Group B (a daily dose of myoinositol: 4000 mg and folic acid: 400 mcg). They evaluated oocyte and embryo quality, clinical pregnancy, and implantation rates. Patients take inositols from the first day of the cycle until 14 days after embryo transfer. They observed that myoinositol and melatonin enhance, synergistically, oocyte and embryo quality so these two can be integrated routinely in association with drugs for ovarian stimulation during IVF cycle [30].

It is well known that ovarian hyperstimulation syndrome (OHSS) is an important iatrogenic complication in ART and that PCOS patients are at an extreme risk for the development of OHSS [31]. Recent trials point out that MYO is effective in preventing OHSS in a way similar to metformin [32].

## 5. Inositol in Non-PCOS Patients in IVF

Few data are available, at the moment, about the effects of MYO supplementation on in vitro fertilization outcome in sterile patients not affected by polycystic ovary syndrome. Recently, Lisi et al. have examined the effects of inositol administration on oocyte and embryo quality in infertile women undergoing IVF cycle by conventional IVF or intracytoplasmic sperm injection (ICSI). One hundred non-PCOS patients aged under 40 years and with basal FSH < 10 mUI/ml undergoing ovarian stimulation are randomly divided into two groups: Group A treated with 400 *μ*g of folic acid for the 3 months before and during rFSH administration and Group B treated with a daily dose of 4000 mg of myoinositol into two administrations/day in addition to 400 *μ*g of folic acid for the 3 months before and during rFSH administration. Group B shows a reduction in the number of mature oocytes retrieved and in the amount of gonadotropins used, whereas implantation rate and clinical pregnancy rate are improved [4].

The effect of MYO supplementation on ovarian function has also been evaluated in poor responders patients [33] undergoing ICSI. The study involves 76 poor responders divided into two groups: 38 patients who have been assuming MYO (4 g) plus folic acid (400 *μ*g) for the previous 3 months before the start (Group A) and 38 patients assuming only folic acid (FA) (400 *μ*g) for the same period (Group B). Ovarian stimulation is carried out with a GnRH antagonist protocol in both groups. They do not observe any significant difference between the two groups regarding oestradiol level, but total rec-FSH units used are significantly lower (*p* = 0.004) and metaphase II (MII) oocytes rate is significantly higher (*p* = 0.01) in Group A. The ovarian sensitivity index is higher, reaching a statistical significance (*p* < 0.05), in the group of patients pretreated with MYO, showing an improvement in ovarian sensibility to gonadotropin. In conclusion, they suggest that MYO supplementation in poor responder patients results in an increase of the number of oocytes recovered in MII and of the gonadotropin ovarian sensitivity, suggesting a MYO role in improving ovarian response to gonadotropins. Hence, MYO seems to be helpful in poor responders undergoing IVF cycles [34].

## 6. Inositol in Sperm Cell

About the role of MYO in male reproduction, Chauvin and Griswold show that MYO concentration in the seminiferous tubules is higher than in serum [35]; moreover, MYO levels are increased by movement of spermatozoa through epididymis and deferent duct [36]. Patients affected by oligoasthenoteratospermia have spermatozoa totally covered by “amorphous fibrous material,” which reduces sperm mobility. Colone et al. show that MYO administration could help to reduce the presence of this amorphous material [37]. MYO has also a crucial role in the osmoregulation of seminal fluid and as a consequence in sperm progressive motility and velocities [38]. Gulino et al. investigate the effect of MYO administration on semen parameters of male patients undergoing IVF cycles. They collect semen samples of 62 patients divided into three different groups: healthy fertile patients (Group A); patients with oligoasthenospermia (OA) (Group B); and control group (CTR). The first two groups receive administration of 4000 mg/die of MYO and 400 *µ*g/die of folic acid for 2 months. Semen's volume and spermatozoa's number and motility are the parameters evaluated before and after treatment and before and after density-gradient separation. Spermatozoa concentrations are higher in both Groups A and B. In conclusion, they showed that MYO supplementation significantly improves semen's parameters both in patients with OA and in normal fertile men [5].

## 7. Conclusion

Nowadays, many studies demonstrate the positive effects of myoinositol in patients undergoing IVF cycle so it could be a predictive factor in improving ART outcomes. In particular, as revealed by a conference scientific committee, MYO improves both ovarian response to gonadotropins during IVF stimulation and oocyte and embryo quality.
