# Corrigendum to “Inositol and In Vitro Fertilization with Embryo Transfer”

**DOI:** 10.1155/2019/8309405

**Published:** 2019-04-11

**Authors:** G. Simi, A. R. Genazzani, M. E. R. Obino, F. Papini, S. Pinelli, V. Cela, P. G. Artini

**Affiliations:** Division of Obstetrics and Gynecology Oncology, Department of Clinical and Experimental Medicine, University of Pisa, Pisa, Italy

In the article titled “Inositol and In Vitro Fertilization with Embryo Transfer” [1], the “Conflicts of Interest” section should read as follows:

“Publication was sponsored by Studio Medico Ass. Agunco.”
